# Gut Microbiota and Liver Health: Meta-Analysis of *Bifidobacterium*-Containing Probiotics in NAFLD Management

**DOI:** 10.3390/ijms26135944

**Published:** 2025-06-20

**Authors:** Ko-Shih Chang, Wu-Hsien Kuo, Mu-Hsin Chang, Yao Hsiao, Ru-Yin Tsai

**Affiliations:** 1 Department of Cardiology, Yuan Rung Hospital, Yuanlin, Changhua 51045, Taiwan; yrh2702@gmail.com; 2 Department of Gastroenterology and Hepatology, Yuan Sheng Branch, Yuan-Rung Hospital, Changhua 51052, Taiwan; wuhsienku@gmail.com; 3 Division of Cardiovascular Medicine, Yuan Rung Hospital, Yuanlin, Chenghua 51045, Taiwan; muhsin.chang@msa.hinet.net; 4 School of Medicine, Chung Shan Medical University, Taichung 40201, Taiwan; s1001085@gm.csmu.edu.tw; 5 Department of Anatomy, School of Medicine, Chung Shan Medical University, Taichung 40201, Taiwan; 6 Department of Medical Education, Chung Shan Medical University Hospital, Taichung 40201, Taiwan

**Keywords:** inflammation, prebiotic, lipid, body mass index, cytokine

## Abstract

Diseases, including cardiovascular disease, type II diabetes, and metabolic syndrome, are leading causes of morbidity and mortality worldwide. Non-alcoholic fatty liver disease (NAFLD) is commonly associated with these conditions through shared pathophysiological mechanisms such as insulin resistance, chronic inflammation, and dyslipidemia. Emerging evidence suggests that probiotic formulations containing Bifidobacterium species may support cardiometabolic health by modulating gut microbiota composition. This meta-analysis aimed to assess the efficacy of Bifidobacterium-containing probiotic combinations in improving key cardiometabolic parameters, including lipid profiles, blood pressure, glycemic indices, and inflammatory biomarkers among individuals with NAFLD. A systematic literature search was conducted across PubMed, Embase, the Cochrane Library, and Web of Science databases to identify relevant randomized controlled trials (RCTs) published up to December 2024. A total of 24 RCTs involving 1611 participants met the inclusion criteria. The pooled results demonstrated significant reductions in total cholesterol, triglycerides, and low-density lipoprotein cholesterol following probiotic intervention. Improvements were also observed in fasting glucose levels and inflammatory markers, including high-sensitivity C-reactive protein (hs-CRP), tumor necrosis factor-alpha (TNF-α), and interleukin-6 (IL-6). Although modest improvements were noted in NAFLD severity, the effects on liver injury markers were relatively limited. These findings suggest that Bifidobacterium-based probiotic combinations may provide cardiometabolic benefits, particularly in lipid regulation, glucose metabolism, and inflammatory control. Further large-scale, well-designed RCTs are warranted to validate these results and to determine the most effective probiotic strains, compositions, and treatment durations.

## 1. Introduction

Cardiometabolic risk factors are widely recognized as predictors of chronic diseases and remain a leading cause of mortality worldwide [1]. These factors are associated with a cluster of metabolic abnormalities, including glucose intolerance, elevated insulin levels, insulin resistance, hypertriglyceridemia, reduced levels of high-density lipoprotein (HDL), and increased concentrations of low-density lipoprotein (LDL). Such imbalances in metabolic regulation contribute to systemic inflammation, as indicated by elevated levels of high-sensitivity C-reactive protein (hs-CRP) and other pro-inflammatory biomarkers [2,3]. Among these markers, both CRP and tumor necrosis factor-alpha (TNF-α) have been identified as important indicators of cardiovascular mortality, including in cases of coronary artery disease [4]. Obesity is another critical component of metabolic syndrome, often reflected in elevated body mass index (BMI), increased waist circumference, and central fat accumulation, all of which significantly raise the risk of developing cardiovascular diseases (CVDs) [5]. Non-alcoholic fatty liver disease (NAFLD), defined by excessive lipid accumulation in liver cells, is increasingly recognized as a hepatic manifestation of metabolic syndrome. It is closely associated with cardiometabolic disorders and related complications [2,6]. Epidemiological data indicate that approximately 25 percent of the global population is affected by NAFLD, with particularly high prevalence seen among individuals with obesity and features of metabolic syndrome [7]. The connection between NAFLD and cardiometabolic conditions stems from shared pathophysiological mechanisms, including insulin resistance, dyslipidemia, systemic inflammation, and cellular stress in the endoplasmic reticulum. These mechanisms collectively promote liver fat accumulation and elevate cardiovascular risk [6,7]. Evidence also indicates that NAFLD is independently associated with an increased risk of cardiovascular disease, beyond traditional risk factors, through pathways involving oxidative stress and chronic inflammation that impair endothelial function and promote atherogenesis [2,8]. NAFLD represents a spectrum of liver disorders ranging from simple steatosis to non-alcoholic steatohepatitis (NASH), which may progress to advanced stages such as fibrosis, cirrhosis, or hepatocellular carcinoma [2]. Despite its increasing prevalence and serious clinical implications, NAFLD lacks approved pharmacological therapies. Current management guidelines emphasize lifestyle modification, including dietary changes and increased physical activity, as the main strategies for disease prevention and control [9].

The gut–liver axis plays a central role in the pathogenesis and advancement of non-alcoholic fatty liver disease (NAFLD), highlighting the dynamic interaction between the gut microbiota and liver metabolism. An altered gut microbial environment, known as a state of dysbiosis, compromises the integrity of the intestinal barrier. This impairment facilitates the passage of bacterial endotoxins, particularly lipopolysaccharides (LPS), into the portal vein. Once in the liver, these endotoxins stimulate inflammatory responses and contribute to the development of insulin resistance, both of which are key contributors to the progression of NAFLD [10,11].

Growing evidence supports the influential role of gut microbiota in regulating both cardiometabolic and liver-related health. Among the beneficial microbial genera, *Bifidobacterium* has been widely studied for its capacity to restore microbial homeostasis and reinforce the integrity of the intestinal barrier. Several studies have demonstrated that *Bifidobacterium* species contribute to the regulation of lipid metabolism, glucose homeostasis, and inflammatory pathways, which are closely associated with the development of NAFLD and cardiometabolic disorders [12,13]. Supplementation with probiotics containing *Bifidobacterium* has been shown to enhance barrier function in the gut epithelium [14], decrease circulating endotoxins, and positively influence lipid metabolism through mechanisms involving the gut–liver axis [15]. Moreover, *Bifidobacterium* has been reported to reduce levels of LPS and inhibit the hepatic activation of toll-like receptor 4 (TLR4), thereby attenuating hepatic inflammation and oxidative stress [16,17]. Additional findings suggest that *Bifidobacterium* may also support bile acid metabolism, which plays a crucial role in lipid regulation and reducing hepatic fat accumulation, offering therapeutic promise in the context of NAFLD [18,19].

This meta-analysis was conducted to assess the effectiveness of *Bifidobacterium* supplementation in improving key cardiometabolic risk parameters, such as lipid profile, blood pressure, glycemic regulation, and inflammatory biomarkers. By synthesizing evidence from randomized controlled trials, the study aimed to determine whether *Bifidobacterium* could function as a supportive therapeutic option for mitigating cardiometabolic risk, especially in individuals with NAFLD and related metabolic disorders.

## 2. Methods

This meta-analysis is registered with PROSPERO (registration number: CRD42024605844) and was conducted in accordance with the PRISMA reporting guidelines [20]. 

### 2.1. Data Sources and Selection Criteria

A comprehensive search was performed across PubMed, Embase, the Cochrane Library, and Web of Science to identify relevant RCTs published up to December 2024. Search terms included combinations such as “*Bifidobacterium*”, “probiotics”, “cardiometabolic”, “lipid profile”, “fatty liver”, “non-alcoholic fatty liver disease”, “non-alcoholic steatohepatitis”, and “steatohepatitis.” In addition, the reference lists of retrieved articles were manually screened to identify further eligible studies. Exclusion criteria included case reports, technical reports, conference abstracts, review articles, editorials, letters, and in vitro or animal-based research.

### 2.2. Selection of Studies

**Figure 1:**
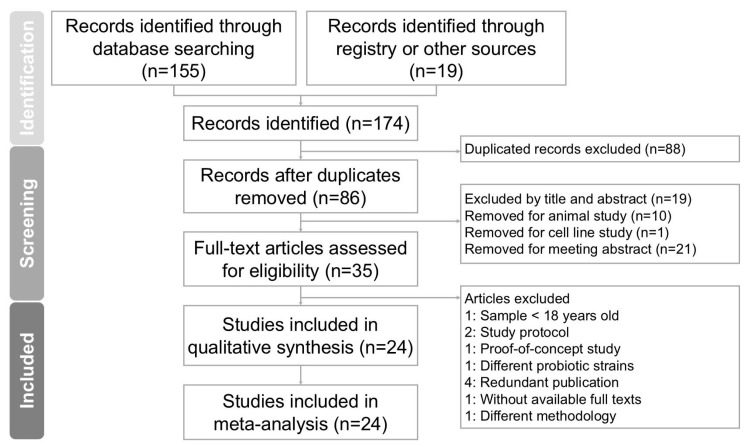
Diagram outlining study selection process for systematic review and meta-analysis investigating *Bifidobacterium* combinations interventions for reducing cardiometabolic risk factors in NAFLD. Of 174 records initially identified, 24 studies fulfilled inclusion criteria and were incorporated into final analysis.

Two independent researchers conducted the screening and evaluation of eligible studies, with a third researcher reviewing the process to maintain consistency and accuracy. Hard copies of all relevant publications were obtained and thoroughly reviewed to ensure detailed assessment. The complete study selection process is outlined in the PRISMA flow diagram (Figure 1).

### 2.3. Data Extraction

Data extraction was independently conducted by authors using a standardized form following the Cochrane Handbook guidelines [20]. Key information collected included author names, publication year and country, participant inclusion criteria, demographic details (such as total sample size and age range), study design, intervention specifics, and reported outcomes, along with the assessment methods used.

### 2.4. Outcomes

The primary outcomes assessed in this study were liver fat content and liver injury markers, including alanine aminotransferase (ALT), aspartate aminotransferase (AST), and gamma-glutamyl transferase (GGT). Inflammatory cytokines such as tumor necrosis factor alpha (TNF-α), hs-CRP, and IL-6 were also evaluated. Additionally, BMI and lipid profile parameters including cholesterol, triglycerides, LDL, and HDL were analyzed. The secondary outcomes included fasting plasma glucose, fasting serum insulin, homeostasis model assessment for insulin resistance (HOMA-IR), glycated hemoglobin (HbA1c), and blood pressure.

### 2.5. Assessment of Methodological Quality

Two independent reviewers systematically evaluated the risk of bias in the included studies using the Cochrane Collaboration’s Risk of Bias tool to assess methodological quality. Any discrepancies between the reviewers were resolved through in-depth discussion with a third reviewer to reach consensus. Studies were deemed to have a high risk of bias if at least one domain was found to raise significant concerns, in accordance with the criteria established by the tool.

### 2.6. Data Analysis

Data from the selected studies were quantitatively analyzed using SMD with 95% confidence intervals (CIs) to compare outcomes between intervention and control (placebo) groups. SMD values were pooled using a random-effects model to account for interstudy variability. Statistical analyses were performed using Comprehensive Meta-Analysis software, version 3 (Version 3.0; Biostat, Englewood, NJ, USA). Heterogeneity was assessed using the *I*^2^ statistic, with values exceeding 50% indicating substantial heterogeneity. Publication bias was evaluated using funnel plots and Egger’s regression test, with significance thresholds set at *p* < 0.05 for general analyses and *p* < 0.10 for publication bias assessment. Subgroup analyses were conducted to investigate the possible sources of heterogeneity, and sensitivity analyses were performed by excluding individual studies to evaluate the stability of the overall results.

## 3. Results

Study search and characteristics of included patients.

The process of screening and selecting trials is illustrated in Figure 1. An initial database search using PubMed, Embase, the Cochrane Library, and Web of Science identified 174 records. After removing duplicate entries, 86 articles remained and were screened based on their titles and abstracts. Of these, 51 articles were excluded due to irrelevance to the research topic. A detailed full-text review was conducted on the remaining 35 articles. Following this review, 11 studies were excluded for the following reasons: one study included participants younger than 18 years [21], two articles were study protocols [22,23], one article was a proof-of-concept study [24], one study used different probiotic strains from the target interventions [25], and study one applied a different methodology [26]. Four studies were excluded due to duplicate publication [27,28,29,30], and one article was excluded due to the full text being unavailable [31]. A total of 24 randomized controlled trials met the eligibility criteria and were included in the meta-analysis [12,13,32,33,34,35,36,37,38,39,40,41,42,43,44,45,46,47,48,49,50,51,52,53]. All selected articles were published in English. Table 1 summarizes the characteristics of the included trials, which were conducted between 2012 and 2024. These studies involved a combined total of 1611 participants, with individual trial sample sizes ranging from 17 to 70. Each study evaluated the effects of *Bifidobacterium* supplementation on body mass index, liver function parameters, and lipid profile outcomes.

### 3.1. Quality Assessment

**Figure 2:**
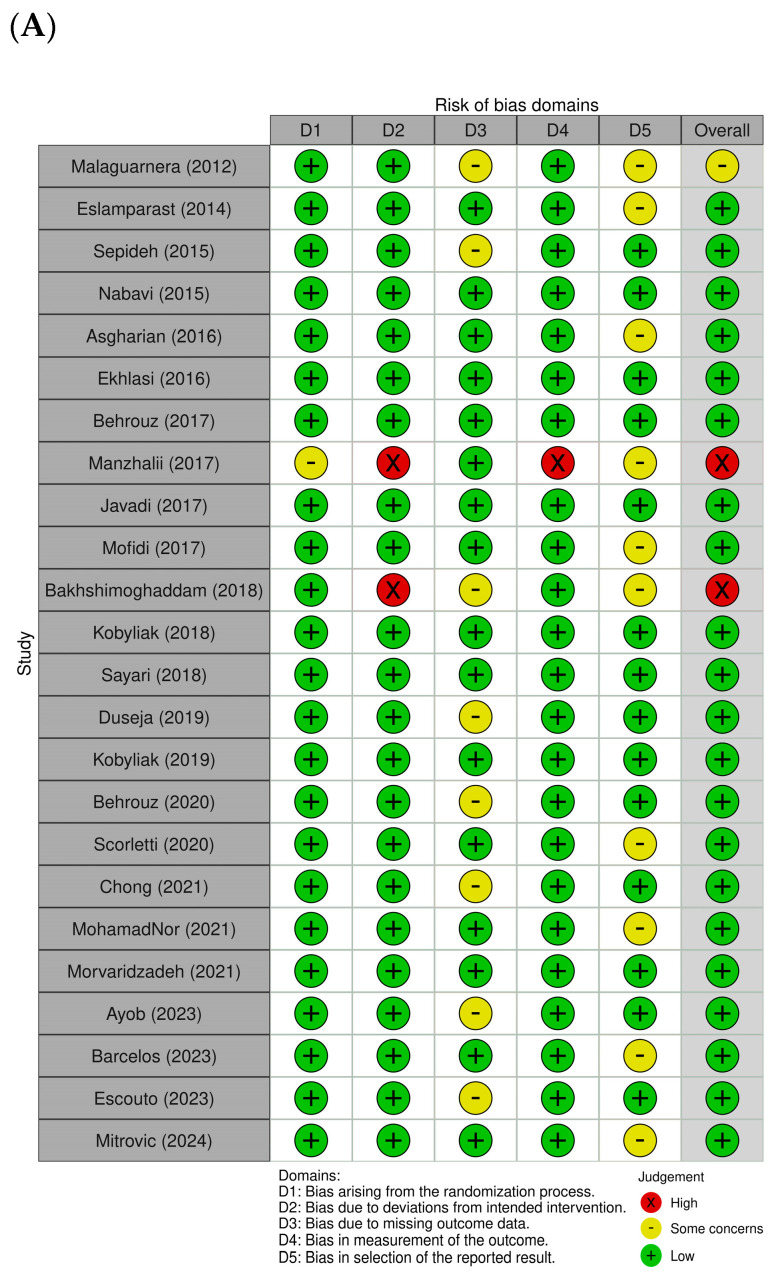

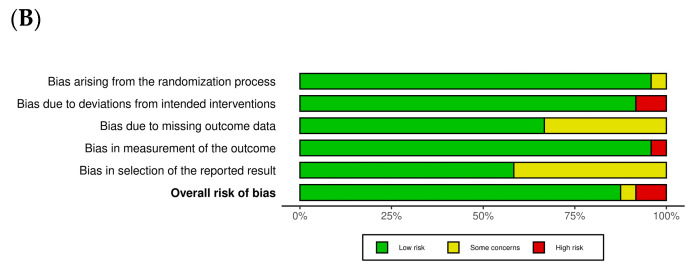
Assessment of methodological quality of included trials. (**A**) Risk of bias for each included study. (**B**) Overall summary of bias of 24 studies [12,13,32,33,34,35,36,37,38,39,40,41,42,43,44,45,46,47,48,49,50,51,52,53].

**Figure 3:**
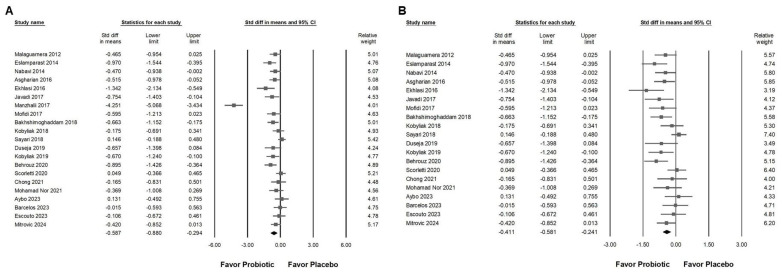
Forest plots show the effects of *Bifidobacterium* combination supplementation on serum ALT levels. Panel (**A**) illustrates the direct impact of the intervention, while Panel (**B**) presents a sensitivity analysis to validate the findings in Panel (**A**). Squares represent standardized mean differences, favoring *Bifidobacterium* (shifted to the left), with horizontal lines indicating 95% confidence intervals. The diamond at the bottom of each plot represents the overall combined effect size for the analysis [12,13,33,38,39,41,43,44,46,47,48,49,50,52,53].

Figure 2A,B provide a summary of the risk of bias assessments across the studies included. Of the trials analyzed, 21 were assessed as having a low risk of bias, reflecting overall methodological strength and clarity in data reporting. Two studies [39,41] exhibited high risk in specific domains, particularly in relation to the accuracy of outcome assessment and fidelity to the intervention protocol. Only one study [41] demonstrated some concern in the randomization process. In domain 2, most trials were rated as displaying low risk, indicating that the interventions were generally delivered as planned, although both studies [39,41] were exceptions, with high risk in this area. Domain 3 presented a recurring issue, with several studies [13,33,35,39,45,46,48,49] rated as displaying low risk, while others showed concerns due to significant missing data. Overall, outcome measurement was consistently and appropriately conducted, with only one trial [41] showing high risk in this domain. Additionally, ten studies raised concerns regarding selective reporting due to the lack of a pre-registered protocol or predefined statistical analysis plan [12,32,33,36,39,41,42,50,52,53]. As part of our quality assessment, we also conducted a sensitivity analysis excluding studies with a high risk of bias. The results remained consistent, indicating that the primary outcomes were robust and not significantly influenced by lower-quality studies. As part of our quality assessment, we also conducted a sensitivity analysis, excluding studies with a high risk of bias. The results remained consistent, indicating that the primary outcomes were robust and not significantly influenced by lower-quality studies (Figure 3A,B).

### 3.2. Effect of Combinations with Bifidobacterium on Alanine Aminotransferase Expression

**Figure 4:**
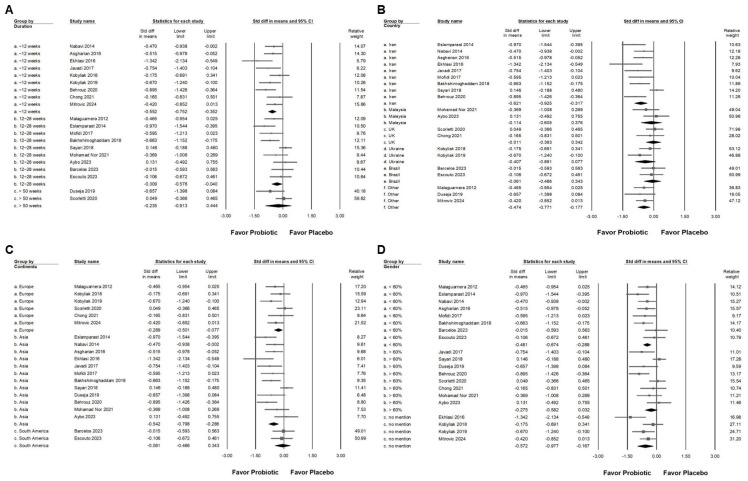
Forest plots summarizing subgroup analyses of *Bifidobacterium* combination supplementation on serum ALT levels. Panel (**A**) evaluates effects by intervention duration, Panel (**B**) by country, and Panel (**C**) by continent. Panel (**D**) examines the influence of proportion of males in intervention groups. Squares indicate standardized mean differences favoring *Bifidobacterium* (left), with horizontal lines showing 95% confidence intervals. Diamonds represent overall combined effect sizes for each analysis [12,13,32,33,34,38,39,40,42,43,44,45,46,47,48,49,50,52,53].

Probiotic combinations that included *Bifidobacterium* demonstrated a moderate effect in reducing serum ALT levels, as presented in Figure 3A (SMD: −0.587, 95% CI: −0.880 to −0.294; *I*^2^ = 83.757%, *p* < 0.001). One particular study [41] reported a substantially greater effect than the others, which influenced its exclusion during sensitivity analysis. After removing this outlier, the updated results (Figure 3B) still demonstrated a statistically significant but smaller effect of *Bifidobacterium* combinations on ALT reduction (SMD: −0.411, 95% CI: −0.581 to −0.241; *I*^2^ = 50.111%, *p* = 0.006). Subgroup analysis based on intervention duration (Figure 4A) indicated that supplementation for 12 weeks resulted in a moderate decrease in ALT (SMD: −0.552, 95% CI: −0.752 to −0.352; *I*^2^ = 18.343%, *p* = 0.280). This effect size was greater compared to that observed in studies with durations of 12 to 28 weeks (SMD: −0.309, 95% CI: −0.578 to −0.040; *I*^2^ = 56.876%, *p* = 0.017) or more than 50 weeks (SMD: −0.235, 95% CI: −0.913 to 0.444; *I*^2^ = 62.344%, *p* = 0.103), both of which showed only a minimal effect. Of the included studies, 42.9 percent were conducted in Iran, raising the possibility that regional or ethnic differences may influence the observed outcomes. Figure 4B illustrates that the impact of *Bifidobacterium* combinations was more pronounced in Iranian participants (SMD: −0.621, 95% CI: −0.925 to −0.317; *I*^2^ = 67.364%, *p* = 0.002) compared to participants from other countries such as Malaysia (SMD: −0.114, 95% CI: −0.605 to 0.376; *I*^2^ = 17.282%, *p* = 0.272), the United Kingdom (SMD: −0.011, 95% CI: −0.363 to 0.342; *I*^2^ = 0%, *p* = 0.593), Ukraine (SMD: −0.407, 95% CI: −0.891 to 0.077; *I*^2^ = 37.186%, *p* = 0.207), and Brazil (SMD: −0.061, 95% CI: −0.466 to 0.343; *I*^2^ = 0%, *p* = 0.827). Further subgroup analysis based on continent of origin (Figure 4C) found that studies conducted in Asia reported a moderate reduction in ALT (SMD: −0.542, 95% CI: −0.796 to −0.286; *I*^2^ = 61.386%, *p* = 0.003). In contrast, studies from Europe demonstrated a smaller effect (SMD: −0.289, 95% CI: −0.501 to −0.077; *I*^2^ = 7.837%, *p* = 0.366), while those from South America showed no significant change (SMD: −0.061, 95% CI: −0.466 to 0.343; *I*^2^ = 0%, *p* = 0.827). Finally, the influence of sex distribution in the intervention groups was assessed (Figure 4D). The results showed no significant difference in terms of the effect of *Bifidobacterium* combinations between groups composed of less than 60 percent males (SMD: −0.481, 95% CI: −0.674 to 0.288; *I*^2^ = 8.492%, *p* = 0.365) and those with more than 60 percent males (SMD: −0.275, 95% CI: −0.582 to 0.032; *I*^2^ = 60.401%, *p* = 0.014), suggesting that the efficacy of the intervention is not substantially influenced by gender composition.

### 3.3. Effect of Combinations with Bifidobacterium on Liver Injury Markers and Liver Fat

**Figure 5:**
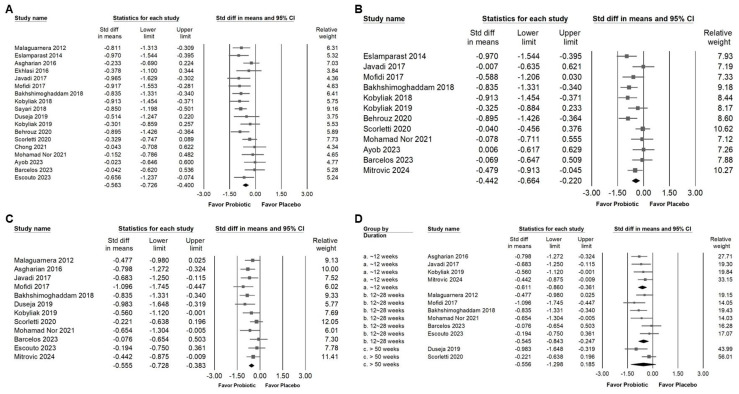
Forest plots illustrate the effects of Bifidobacterium combination supplementation on liver enzymes and liver fat. Panel (**A**) presents results for AST levels, Panel (**B**) presents results for GGT levels, and Panel (**C**) presents results for liver fat content; Panel (**D**) shows a subgroup analysis of liver fat content based on intervention duration. Squares represent effect estimates with horizontal lines showing 95% confidence intervals. Diamonds at the bottom of each panel indicate the overall pooled effect size for each outcome [13,33,38,39,42,43,44,46,47,49,50,52].

*Bifidobacterium* combination supplementation demonstrated a moderate effect in reducing AST levels, as shown in Figure 5A (SMD = −0.563, 95% CI: −0.726 to −0.400; *I*^2^ = 36.3%, *p* = 0.063). A smaller yet significant effect was observed for GGT (Figure 5B; SMD = −0.442, 95% CI: −0.664 to −0.220; *I*^2^ = 50.1%, *p* = 0.024). Additionally, a moderate reduction in liver fat content was observed (SMD = −0.555, 95% CI: −0.728 to −0.383; *I*^2^ = 20.6%, *p* = 0.241; Figure 5C). To further explore the influence of intervention duration, we conducted subgroup analysis (Figure 5D). Moderate effects were maintained in both the <12-week group (SMD = −0.611, 95% CI: −0.860 to −0.361; *I*^2^ < 0.001%, *p* = 0.735) and the 12–28-week group (SMD = −0.545, 95% CI: −0.843 to −0.247; *I*^2^ = 40.0%, *p* = 0.139). In contrast, no significant effect was observed in studies lasting over 50 weeks (SMD = −0.556, 95% CI: −1.298 to 0.185; *I*^2^ = 72.4%, *p* = 0.057). These findings suggest that the beneficial effects of *Bifidobacterium* combinations on liver fat may be more pronounced during short- to medium-term interventions.

### 3.4. Influence of Combinations with Bifidobacterium on Blood Lipids and Lipoproteins

**Figure 6:**
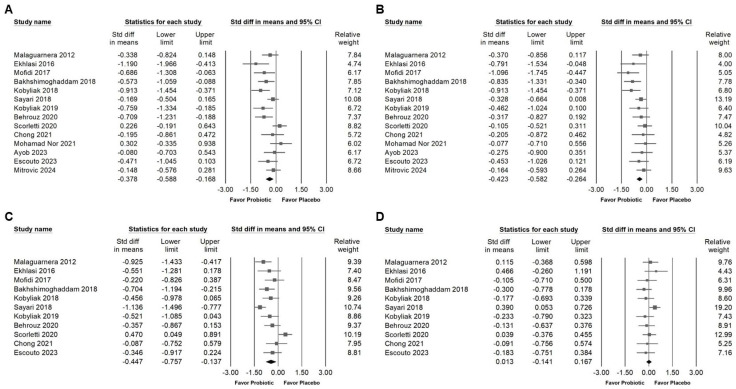
Forest plots illustrating effects of *Bifidobacterium* combination supplementation on lipid profile parameters. Panel (**A**) shows total cholesterol, Panel (**B**) shows triglycerides, Panel (**C**) shows LDL, and Panel (**D**) shows HDL levels. Squares represent standardized mean differences with 95% confidence intervals, while diamonds summarize the overall pooled effect size for each outcome, highlighting the intervention’s impact on lipid metabolism [13,33,38,39,42,43,44,46,47,48,49,50,52].

Supplementation with *Bifidobacterium* combinations demonstrated a small but statistically significant reduction in serum total cholesterol levels, as indicated in Figure 6A (SMD: −0.378, 95% CI: −0.588 to −0.168; *I*^2^ = 54.782%, *p* = 0.007). Similar effects were observed for triglycerides (Figure 6B; SMD: −0.423, 95% CI: −0.582 to −0.264; *I*^2^ = 22.693%, *p* = 0.208) and LDL cholesterol (Figure 6C; SMD: −0.447, 95% CI: −0.757 to −0.137; *I^2^* = 74.125%, *p* = 0). In contrast, no significant change was detected in HDL cholesterol levels (Figure 6D; SMD: 0.013, 95% CI: −0.141 to 0.167; *I*^2^ = 3.937%, *p* = 0.405). These results suggest that *Bifidobacterium* combinations may contribute to improved lipid metabolism, particularly through reductions in atherogenic lipid components such as total cholesterol, triglycerides, and LDL.

### 3.5. Effects of Combinations with Bifidobacterium on Fasting Plasma Glucose, Serum Insulin, HbA1C, and Homeostasis Model Assessment for Insulin Resistance (HOMA-IR)

**Figure 7:**
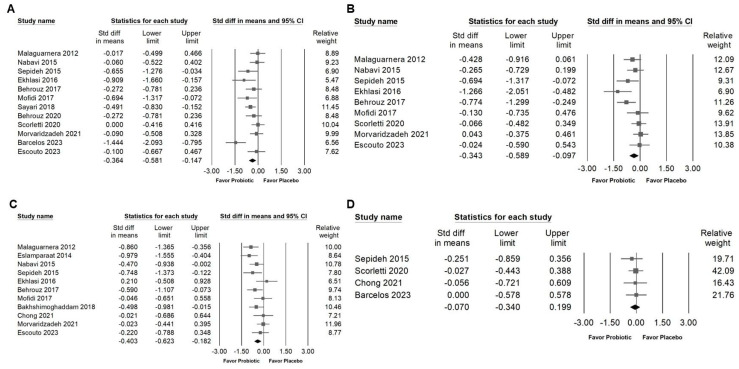
Forest plots summarizing the effects of *Bifidobacterium* combination supplementation on glycemic control markers. Panel (**A**) shows fasting blood glucose, Panel (**B**) shows fasting serum insulin, Panel (**C**) shows HOMA-IR, and Panel (**D**) shows HbA1C levels. Squares indicate effect estimates with 95% confidence intervals, and diamonds at the bottom of each panel represent the overall pooled effect size, illustrating the intervention’s impact on glycemic markers [13,33,34,35,37,38,42,44,46,51,52,53].

Supplementation with probiotic combinations containing *Bifidobacterium* was associated with a small but significant reduction in fasting blood glucose levels (Figure 7A; SMD: −0.364, 95% CI: −0.581 to −0.147; *I*^2^ = 54.496%, *p* = 0.012), serum insulin concentrations (Figure 7B; SMD: −0.343, 95% CI: −0.589 to −0.097; *I*^2^ = 49.413%, *p* = 0.045), and HOMA-IR values (Figure 7C; SMD: −0.403, 95% CI: −0.623 to −0.182; *I*^2^ = 44.094%, *p* = 0.057). In contrast, no significant change was found in glycated hemoglobin (HbA1c) levels (Figure 7D; SMD: −0.070, 95% CI: −0.340 to 0.199; *I*^2^ = 0%, *p* = 0.931). These results suggest that *Bifidobacterium* combinations may contribute to short-term improvements in glycemic regulation, particularly by lowering fasting glucose and enhancing insulin sensitivity, while their influence on long-term glycemic markers remains limited.

### 3.6. Effect of Combinations with Bifidobacterium on Blood Pressure and Inflammatory Cytokines

**Figure 8:**
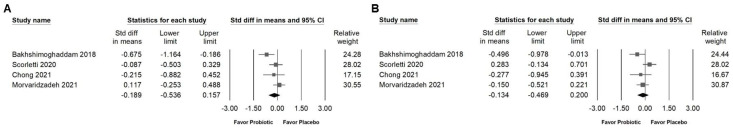
Forest plots summarizing the effects of *Bifidobacterium* combination supplementation on blood pressure. Panel (**A**) shows changes in systolic blood pressure, and Panel (**B**) in diastolic blood pressure. Squares represent effect estimates with 95% confidence intervals, and diamonds indicate the overall pooled effect size, highlighting the intervention’s impact on blood pressure [39,49,51,52].

**Figure 9:**
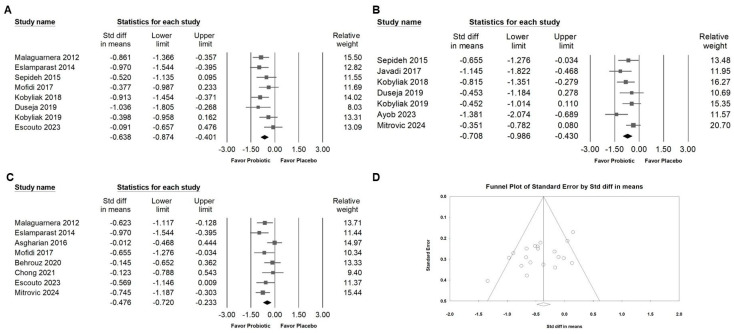
Forest plots summarizing the effects of *Bifidobacterium* combination supplementation on inflammatory markers. Panel (**A**) examines TNF-α, Panel (**B**) shows IL-6, and Panel (**C**) shows hs-CRP levels. Squares represent effect estimates with 95% confidence intervals, and diamonds indicate the overall pooled effect size. Panel (**D**) shows a funnel plot for the studies in Figure 3B; the lines represent the confidence intervals around the effect estimates, indicating the range within which the true effect size is likely to fall. Each circle corresponds to an individual study included in the meta-analysis, with the size of the circle potentially reflecting the study’s weight or sample size. Larger circles denote studies with greater weight or larger sample sizes. The diamond symbol signifies the overall effect estimate from the meta-analysis. The center of the diamond marks the pooled effect size, and the width of the diamond indicates the confidence interval for this estimate [13,33,38,39,42,43,44,46,47,49,50,52].

The meta-analysis indicated that *Bifidobacterium* combinations did not significantly influence systolic blood pressure (Figure 8A; SMD: −0.189, 95% CI: −0.536 to 0.157; *I*^2^ = 54.257%, *p* = 0.087) or diastolic blood pressure (Figure 8B; SMD: −0.134, 95% CI: −0.469 to 0.200; *I*^2^ = 51.150%, *p* = 0.105). In contrast, supplementation with *Bifidobacterium* combinations was associated with a significant reduction in pro-inflammatory cytokines, including TNF-α (Figure 9A; SMD: −0.638, 95% CI: −0.874 to −0.401; *I*^2^ = 23.801%, *p* = 0.240), IL-6 (Figure 9B; SMD: −0.708, 95% CI: −0.986 to −0.430; *I*^2^ = 35.094%, *p* = 0.160), and hs-CRP (Figure 9C; SMD: −0.476, 95% CI: −0.720 to −0.233; *I*^2^ = 40.249%, *p* = 0.110), suggesting a potential anti-inflammatory benefit despite the limited impact on blood pressure parameters.

### 3.7. Publishing Bias

Funnel plots illustrating the SMD for the effectiveness of *Bifidobacterium* combination interventions in reducing ALT levels are presented in Figure 7D. As shown in Figure 2B, Egger’s regression analysis suggested the presence of potential publication bias, with a *p*-value of 0.01574.

## 4. Discussion

This meta-analysis examined the impact of *Bifidobacterium*-containing probiotic combinations on metabolic and hepatic outcomes, including lipid profiles, glycemic control, inflammatory cytokines, and liver injury markers. Significant improvements were observed, particularly in lipid modulation and inflammation attenuation. However, the effect sizes varied according to supplementation duration and population characteristics.

Our findings indicated that probiotic combinations containing *Bifidobacterium* produced a moderate reduction in liver fat content and exerted a notable influence on lipid metabolism, particularly by decreasing levels of total cholesterol, triglycerides, and LDL. These outcomes are consistent with earlier research suggesting that probiotics may regulate lipid metabolism through mechanisms such as bile salt deconjugation and cholesterol assimilation by gut microbiota [54]. In contrast, HDL levels exhibited minimal change in response to Bifidobacterium supplementation. This disparity suggests that probiotics may be more effective in lowering harmful lipid fractions than in promoting beneficial ones. The relatively stable HDL concentrations may stem from the unique physiological role of HDL in reverse cholesterol transport—a process less susceptible to modulation by dietary and microbial interventions compared to LDL and triglyceride pathways [55]. Probiotic action, particularly from Bifidobacterium, appears to target cholesterol metabolism by altering bile acid composition, decreasing intestinal cholesterol absorption, and increasing fecal lipid excretion. These mechanisms predominantly influence LDL and triglyceride levels. Consequently, Bifidobacterium combinations may offer a promising adjunctive approach for managing dyslipidemia. The concurrent reduction in BMI observed across studies is clinically relevant, given the strong association between obesity and NAFLD. Weight reduction remains a primary strategy in NAFLD management, and even modest decreases in BMI have been linked to reductions in hepatic fat accumulation and improvements in systemic metabolic markers, thereby lowering the risk of disease progression [56,57].

The intervention was associated with modest improvements in glycemic regulation, as evidenced by reductions in fasting blood glucose, serum insulin, and the homeostatic model assessment for insulin resistance (HOMA-IR). However, no significant changes were detected in HbA1c levels—a finding that aligns with previous studies [58,59]. The lack of improvement in HbA1c, a marker of long-term glycemic status, suggests that these probiotic interventions may not sustain glucose-lowering effects over extended periods. This disparity may reflect the time-limited nature of probiotic influence on insulin signaling pathways or the requirement for prolonged intervention to achieve durable metabolic benefits. As such, while Bifidobacterium combinations appear to offer supplementary benefits in early glycemic control, particularly for individuals with insulin resistance or prediabetes, future research involving longer intervention durations and longitudinal monitoring is warranted to confirm their long-term efficacy.

An important observation from this meta-analysis was the moderate reduction in hepatic enzyme levels, particularly ALT and AST, following supplementation with Bifidobacterium combinations. In contrast, reductions in GGT were less pronounced. Notably, ALT levels declined more substantially in trials with shorter intervention periods of around 12 weeks, suggesting that the hepatoprotective effects may reach a therapeutic plateau over time [60]. This finding is clinically relevant, as elevated ALT and AST are well-established biomarkers of hepatocellular injury, especially in individuals with NAFLD. Their reduction implies a decrease in hepatic inflammation and injury, indicating improved liver health [61]. The beneficial effect on liver enzymes may be attributable to the ability of Bifidobacterium to modulate the gut–liver axis by restoring microbial balance, reinforcing intestinal barrier integrity, and reducing the translocation of endotoxins such as lipopolysaccharides. These actions help dampen systemic inflammation and alleviate hepatic stress. Moreover, Bifidobacterium has been shown to regulate bile acid metabolism and mitigate oxidative stress, mechanisms that are critical in hepatocyte injury and regeneration [54]. These multifaceted pathways likely contribute to the greater responsiveness of ALT and AST compared to systemic markers like HbA1c or HDL, which may require longer durations to exhibit significant changes due to their association with chronic metabolic regulation.

Subgroup analysis indicated that Bifidobacterium combination supplementation significantly reduced ALT levels in studies where the proportion of males in the intervention group was less than 60%. This outcome suggests that the intervention may have a more pronounced effect in female participants, potentially reflecting gender-specific sensitivity to probiotic therapy. One possible explanation lies in sex-based differences in gut microbiota composition. Women have been reported to exhibit a higher Firmicutes-to-Bacteroidetes (F/B) ratio compared to men, a microbial profile that may influence how probiotics like Bifidobacterium interact with the host’s intestinal ecosystem [62]. Additionally, sex hormones such as estrogen play a modulatory role in shaping the gut microbiota, potentially affecting how females respond to probiotic supplementation [63]. Despite these insights, few studies have specifically explored the relationship between gender and changes in ALT levels following probiotic use. Some evidence suggests gender-dependent differences in microbial responses to metabolic stimuli. For instance, one study showed that sex-specific gut microbiota patterns are associated with variations in metabolic risk and liver enzyme levels [64]. Another investigation highlighted how age and gender may influence the clinical efficacy of probiotics, especially regarding metabolic outcomes [65]. However, a systematic evaluation of sex as a modifying factor in probiotic trials is still lacking. Moreover, differences in probiotic strain composition, dosage, and duration across the included studies may further confound subgroup analyses. Therefore, future research should aim to incorporate standardized intervention protocols and stratify outcomes by gender to clarify the extent to which probiotic efficacy differs between sexes and to inform precision-based therapeutic strategies.

This meta-analysis highlights the potential of *Bifidobacterium*-containing probiotics in addressing NAFLD-related risk factors; however, several limitations warrant consideration. First, significant heterogeneity was observed across the studies included, likely stemming from differences in probiotic strains, combinations with other genera, dosing regimens, and the duration of the interventions. These variations hinder direct comparison and reduce the precision of the overall effect estimates. Although subgroup and sensitivity analyses were conducted, not all sources of heterogeneity could be fully explained. Second, Egger’s regression test indicated potential publication bias (*p* = 0.01574), suggesting that studies with null or unfavorable outcomes may be underrepresented in the literature. This raises the possibility that the observed benefits could be overstated due to selective reporting. Third, the duration of most included trials was relatively short, typically ranging from 8 to 12 weeks. Although nine studies had follow-up periods between 12 and 28 weeks and demonstrated a sustained, albeit smaller, effect (SMD = −0.309), longer-term outcomes remain uncertain. In particular, key endpoints such as HbA1c, HDL cholesterol, histological liver changes, and progression of NAFLD were either minimally affected or not assessed, limiting the ability to determine the durability and clinical significance of the interventions. Fourth, nearly half of the included trials (12 out of 24) were conducted in Iran. This regional concentration may restrict the generalizability of the findings, as dietary habits, lifestyle factors, and genetic backgrounds can significantly influence gut microbiota composition and responsiveness to probiotics. Moreover, some studies may involve overlapping participant populations from the same institutions, raising concerns about potential data redundancy and inflation of pooled effects. Lastly, none of the included trials systematically accounted for habitual dietary intake or host genetic factors—both of which are known to modulate the gut microbiome and may interact with probiotic efficacy. Without controlling for these variables, the interpretation of the observed benefits across diverse populations remains limited. To enhance future research, well-designed, multicenter randomized controlled trials with longer durations are needed. These studies should incorporate standardized probiotic formulations, stratify factors such as diet and genetics by host, and include clinically meaningful endpoints to better assess the long-term efficacy and applicability of probiotic interventions.

## 5. Conclusions

In conclusion, while current evidence does not support the reversal of NAFLD by *Bifidobacterium* combinations, this meta-analysis suggests their potential in improving key metabolic and inflammatory markers, such as LDL, triglycerides, glucose, insulin, TNF-α, IL-6, and hs-CRP. These combinations may serve as adjuncts to lifestyle changes. Larger, standardized RCTs focused on diverse populations are needed to confirm long-term benefits and clinical applicability.

## Figures and Tables

**Table 1:** Characteristics of included studies.

Author (Year)/Country	Inclusion Criteria	Exclusion Criteria	Sample Size (% of Male)/Age	Study Design	Placebo Using	Intervention/Duration	Main Results	Secondary Results
8 Weeks								
Nabavi (2015) [34]/Iran	With NAFLD, aged 23–63, BMI between 25–40 kg/m^2^, and ultrasound-confirmed NAFLD.	Kidney disease, other liver diseases (e.g., hepatitis B, C), thyroid disorders, inflammatory bowel disease, immune deficiency diseases, pregnancy, breastfeeding, use of specific medications (cholesterol-lowering drugs, estrogen, progesterone, or diuretics), recent use of probiotics or probiotic yogurt within the last two months.	P: 36 (50) I: 36 (48.2)/ P: 44.05 ± 8.14 I: 42.75 ± 8.72	RCT/double-blind/placebo	Conventional yogurt as control (no probiotic strains added).	300 g/day of probiotic yogurt (*containing Lactobacillus acidophilus La5* and *Bifidobacterium lactis Bb12*).	Reductions in BMI and fasting insulin levels. No differences observed in serum leptin or adiponectin levels.	No change in waist circumference, insulin resistance (HOMA-IR), or leptin-to-adiponectin ratio.
Sepideh (2015) [35]/Iran	Adults diagnosed with NAFLD by ultrasound, aged 18–65.	Alcohol use, thyroid/kidney disease, viral hepatitis, pregnancy, lactation, recent use of probiotics, NSAIDs, or antibiotics within two months prior.	P: 21 (71.4) I: 21 (61.9)/ P: 47.33 ± 2.53 I: 42.10 ± 1.99	RCT/double-blind/placebo	Capsules similar in appearance to probiotic capsules.	Probiotic containing multiple strains (*Lactobacillus acidophilus, Bifidobacterium breve*) two capsules/day.	Reduction in FBS, insulin, HOMA-IR, and TNF-α and IL-6.	No changes in HbA1C levels between groups.
Asgharian (2016) [36]/Iran	1. Aged between 18 and 60 years and diagnosed with NAFLD by ultrasound. 2. Have no other liver diseases, such as hepatitis B or C. 3. No use of corticosteroids or specific medications that influence liver function.	1. Pregnancy or lactation. 2. Use of vitamin-mineral, antioxidant, or omega-3 supplementation. 3. Inability to comply with the study’s protocol (<90% compliance). 4. Use of antibiotics during the study.	P: 36 (35.3) I: 38 (17.5)/ P: 47.78 ± 1.7 I: 46.57.05 ± 1.7	RCT/double-blind/placebo	500 mg capsules containing 120 mg starch	500 mg symbiotic capsule containing: *Lactobacillus casei,* L. *acidophilus*, L. *rhamnosus,* L. *bulgaricus, Bifidobacterium breve, B. longum, Streptococcus thermophilus* and *fructooligosaccharides.*	Improved the ultrasound grade of hepatic steatosis. Significant weight reduction.	No differences in physical activity or dietary intake were observed between the groups. No change in ALT and AST within the symbiotic group, but the placebo group showed a significant increase in both ALT and AST levels. CRP levels remained unchanged in both groups.
Ekhlasi (2016) [38]/Iran	Diagnosed with NAFLD via ultrasound (Grade 1–3) and elevated ALT levels (>30 mg/dL) for at least six months before and at the time of randomization, ages 25–64, BMI 25–35 kg/m^2^.	Liver pathologies like viral hepatitis, alcohol consumption, hypothyroidism, autoimmune diseases, diabetes, cancer history, and certain medication use (NSAIDs, antibiotics, probiotics, supplements).	Placebo: 15 Probiotic: 15 Vitamin E: 15 Probiotic + Vitamin E: 15/ no mention	RCT/double-blind/placebo/four-arm study	Identical placebo capsules (corn starch) for both symbiotic and vitamin E groups.	Symbiotic: Twice daily (*Lactobacillus casei,* L. *rhamnosus, S. thermophilus, B. breve,* etc.). Vitamin E: 400 IU per day.	Combination of symbiotic and vitamin E significantly reduced ALT, AST, ALP, and leptin levels. Significant decreases in TG, TC, and LDL were observed in both the symbiotic and symbiotic + vitamin E groups.	Significant reductions in FBS and insulin levels were observed in the symbiotic + vitamin E group. No significant changes were seen in HDL or HOMA across all groups.
Kobyliak (2018) [43]/Ukraine	Adult participants aged 18–65 years with a BMI > 25 kg/m^2^, diagnosed with NAFLD based on clinical examination, laboratory values, liver enzyme activities (ALT, AST), and ultrasound examination.	Alcohol abuse, chronic viral hepatitis, drug-induced liver disease, Wilson’s disease, hereditary deficiency of antitrypsin-1, idiopathic hemochromatosis, history of decompensated liver disease, regular use of probiotics or prebiotics within 3 months prior to enrollment, antibiotic use within 3 months prior to enrollment, uncontrolled cardiovascular or respiratory disease, active malignancy, chronic infections, use of medications that affect NAFLD, active infection, pregnancy, or lactation.	P: 28 I: 30/ P: 57.29 ± 10.45 I: 53.4 ± 9.55	RCT/double-blind/placebo	A placebo sachet identical in appearance, texture, and weight to the probiotic sachet.	1 sachet (10 g) of multiprobiotic “Symbiter” containing 14 live probiotic strains (*Lactobacillus, Lactococcus, Bifidobacterium, Propionibacterium, Acetobacter*) or placebo daily.	Probiotic intake significantly decreased the FLI, serum levels of AST and GGT, and the levels of inflammatory cytokines TNF-α and IL-6 in NAFLD patients.	Significant reduction in TG, total cholesterol, LDL-C, and VLDL-C levels was observed in the probiotic group compared to the placebo group.
Kobyliak (2019) [47]/Ukraine	Adults with type 2 diabetes (T2D), BMI ≥ 25 kg/m^2^, and NAFLD diagnosed via ultrasound. Patients on stable anti-diabetic medications for at least four weeks prior.	Decompensated liver disease, viral hepatitis, alcohol abuse, recent probiotic, prebiotic, or antibiotic use, recent vitamin E or omega-3 fatty acid supplementation, and active cardiovascular or respiratory disease.	P: 24 I: 26/ P: 57.38 ± 9.92 I: 53.23 ± 10.09	RCT/double-blind/placebo	Sachets with similar taste and appearance as the intervention.	Symbiter Forte sachets (probiotic with smectite gel), containing multiple probiotic strains, administered daily for 8 weeks.	Reductions in LS and ALT levels in the probiotic-smectite group compared to placebo. Reductions in total cholesterol and cytokines (IL-1b and TNF-a) in the intervention group.	Insignificant changes in FLI and some liver enzymes (e.g., AST) compared to placebo. The intervention group showed improvements in systemic inflammation markers.
8~12 weeks								
Javadi (2017) [40]/Iran	Adults aged 20–60 with NAFLD (diagnosed via ultrasound and elevated ALT/AST levels).	Cardiovascular, thyroid, kidney, autoimmune diseases, hepatitis A, B, or C, hemochromatosis, Wilson’s disease, alcohol consumption, pregnancy, and lactation.	Placebo: 19 (68.4) Probiotic: 20 (85) Prebiotic: 19 (84.2) Probiotic + Prebiotic: 17 (82.4)/ Placebo: 42.21 ± 9.11 Probiotic: 43.90 ± 9.02 Prebiotic: 38.68 ± 10 Probiotic + Prebiotic: 43.24 ± 6.95	RCT/double-blind/placebo/four-arm study	Maltodextrin powder for prebiotic placebo and lactose-free milk powder for probiotic placebo.	Probiotic: *Bifidobacterium longum* and *Lactobacillus acidophilus* (2 × 10^7 CFU daily) Prebiotic: Inulin (10 g/day)	Reductions in LDL and increases in HDL in the probiotic and combined groups compared to placebo. Weight and BMI decreased in the intervention groups.	Insulin resistance markers (HOMA-IR, fasting insulin) significantly improved within the combined probiotic and prebiotic group compared to baseline, but no notable changes in glucose levels across groups.
Manzhalii (2017) [41]/Ukraine	1. Age 30–60 years. 2. Diagnosed with NASH based on ultrasonography, elevated ALT and GGT levels, and increased liver stiffness as measured by Fibroscan.	1. Chronic liver diseases (e.g., viral hepatitis, alcoholic steatohepatitis, autoimmune hepatitis). 2. Obesity (BMI > 30 kg/m^2^). 3. Diabetes mellitus (fasting blood glucose > 5.6 mmol/L). 4. Hypertriglyceridemia (>1.7 mmol/L). 5. Severe comorbidities. 6. Pregnant or lactating women.	P: 37 (43.2) I: 38 (28.9)/ P: 43.5 ± 1.3 I: 44.3 ± 1.5	RCT/double-blind/placebo	No placebo was used. Control group received only a low-fat/low-calorie diet without probiotics.	Probiotic cocktail containing: *Lactobacillus casei,* L. *rhamnosus,* L. *bulgaricus, Bifidobacterium longum, Streptococcus thermophilus*, and *fructooligosaccharides.*	Improvements in BMI and total cholesterol levels in the probiotic group. Liver stiffness (measured by Fibroscan) improved significantly in the probiotic group (*p* < 0.05).	Reduction in ALT and AST levels in the probiotic group (*p* < 0.05). No significant reduction in GGT levels. Changes in the fecal microbial composition showed an increase in beneficial bacteria (*Bifidobacteria, Lactobacilli*) and a reduction in pathogenic bacteria (e.g., *Klebsiella, Candida*) in the probiotic group.
Behrouz (2017) [37]/Iran	Adults aged 20–60 with NAFLD, BMI ≥ 25 kg/m^2^, ALT > 1.5 times the upper limit of normal, and liver steatosis greater than grade II on ultrasound.	Alcohol use, pregnancy, lactation, liver conditions (hepatitis B/C, cirrhosis), celiac disease, cardiovascular, kidney, lung diseases, recent use of antibiotics, supplements, NSAIDs, and corticosteroids.	Prebiotic: 29 (69) Placebo: 30 (70) Probiotic: 30 (73.3)/Prebiotic: 38.41 ± 9.21 Placebo: 38.43 ± 10.09 Probiotic: 38.46 ± 7.11	RCT/double-blind/placebo/three-arm study	Maltodextrin capsules.	Probiotic: *Lactobacillus casei,* L. *rhamnosus,* L. *acidophilus, Bifidobacterium longum, and B. breve* (5 billion CFU/day). Prebiotic: *Oligofructose* powder (16 g/day).	Reductions in serum leptin, insulin, FBS, and HOMA.	QUICKI (insulin sensitivity) improved in probiotic and prebiotic groups.
Behrouz (2020) [46]/Iran	Diagnosed with NAFLD (elevated ALT >1.5 × ULN, steatosis grade > II on ultrasound), age 20–60, BMI 25–40 kg/m^2^.	Severe cardiac/renal impairment, other liver disorders, diabetes, hypertension, inflammatory diseases, recent weight-loss diets, regular corticosteroids/NSAIDs, recent antibiotic use, pregnancy, and alcohol abuse.	Prebiotic: 29 (69) Placebo: 30 (70) Probiotic: 30 (73.3)/ no mention	RCT/double-blind/placebo/three-arm study	Maltodextrin capsules.	Probiotic: 5 billion CFU mix (*Lactobacillus casei,* L. *rhamnosus,* L. *acidophilus*, *Bifidobacterium longum, B. breve*) daily. Prebiotic: *Oligofructose* 8 g twice/day.	No change in adiponectin levels.	hs-CRP decreased in all groups, without significant differences between groups. No significant changes in HDL, LDL, HDL ratios, or glucose levels across groups.
Morvaridzadeh (2021) [51]/Iran	Diagnosed NAFLD patients aged 25–55 years with mild to moderate fatty liver confirmed by ultrasonography.	Use of supplements or medications within six weeks before or during the study; sensitivity to yogurt; and presence of other chronic diseases, including heart, lung, kidney disease, or cancer.	P: 52 (54.5) I: 52 (52.2)/ P: 39.91 ± 7.16 I: 40.39 ± 6.22	RCT/double-blind/placebo	Yogurt without Vitamin D fortification	Daily consumption of 100 g of fortified yogurt (intervention) or regular yogurt (control) over 12 weeks. Multi-strain probiotic with Vitamin D fortification.	Significant increase in serum 25(OH)D3 levels. Significant decrease in insulin levels in the intervention group. No significant change in fasting blood sugar levels between groups.	No significant changes in anthropometric measures, such as weight, BMI, waist circumference, or lean body mass.
Chong (2021) [49]/UK	Patients aged 25–70 years with confirmed NAFLD (via biopsy or imaging) and at least a 20% risk of a cardiovascular event over the next 10 years (using an adjusted QRisk2 score).	Established cardiovascular disease, decompensated liver cirrhosis, allergy/intolerance to VSL#3^®^, chronic alcohol intake above set limits, recent or repeated antibiotic use, solid organ or bone marrow transplant, and oral corticosteroid therapy.	P: 16 (81.2) I: 19 (78.9)/ P: 58 ± 7 I: 57 ± 8	RCT/double-blind/placebo	non-identifiable sachets that matched the VSL#3^®^ sachets in appearance, containing an inert substance.	VSL#3^®^ probiotic (8 different strains including *Lactobacillus* and *Bifidobacterium*)	No improvement in insulin resistance, endothelial function, oxidative stress, inflammation, or liver injury markers with VSL#3^®^ compared to placebo.	Positive correlations noted between markers of inflammation and endothelial dysfunction at baseline, but no significant change in NAFLD fibrosis score or other markers post-treatment.
Mitrović (2024) [53]/Serbia	Patients diagnosed with MASLD, defined by an elastometric attenuation coefficient (ATT) greater than 0.63 dB/cm/MHz with an ALT level above 40 U/L for men and 35 U/L for women.	Unwillingness to provide informed consent, alcohol consumption of more than 20 g per day for the 6 months before enrollment, viral hepatitis, liver cancer, bowel or liver resection, previous IBD, hypothyroidism, or the use of different probiotic or antibiotic therapy in the two weeks before and during the study.	P: 43 I: 41/ P: 68 ± 9 I: 69 ± 8	RCT/double-blind/placebo	Identical capsules containing 6.8 g of maltodextrin.	Two capsules containing a total of 64 billion CFU of *Lactobacillus acidophilus*, *Lactobacillus casei*, and *Bifidobacterium lactis*, combined with 6.4 g of inulin.	Synbiotic therapy significantly reduced liver steatosis as measured by ATT and decreased levels of hs-CRP compared to the placebo group. The microbiome analysis showed a significant enrichment in *Lactobacillus*, *Bifidobacterium*, *Faecalibacterium*, and *Streptococcus genera*, along with a reduction in *Ruminococcus* and *Enterobacterium*.	Synbiotics significantly shortened gut transition time and reduced constipation symptoms in MASLD patients. However, the therapy did not show significant effects on liver stiffness (E median), ALT levels, lipid metabolism, HbA1c, or eGFR when compared to the placebo group.
12~24 weeks								
Malaguarnera (2012) [33]/Italy	1. Aged between 30 and 65 years. 2. Diagnosis of NASH confirmed by liver biopsy. 3. Abnormal aminotransferase levels for at least six months. 4. Sonographic findings compatible with hepatic steatosis.	1. Chronic liver diseases (e.g., hepatitis B, hepatitis C, autoimmune liver disease). 2. Prior surgical procedures such as jejunoileal bypass. 3. Decompensated liver disease (ascites, bleeding varices, hepatic encephalopathy). 4. Pregnancy. 5. History of alcohol consumption greater than 10 g/day for females or 20 g/day for males. 6. Use of lipid-lowering agents, steroids, methotrexate, or high-dose synthetic estrogens.	P: 32 (46.9) I: 34 (52.9)/ P: 46.7 ± 5.7 I: 46.9 ± 5.4	RCT/double-blind/placebo	Sachets with placebo (matching *Bifidobacterium longum* with *fructo-oligosaccharides* formulation)	*Bifidobacterium longum* with FOS.	Reductions in liver enzymes (AST and ALT). Reduction in LDL, cholesterol, and CRP (−2.9 vs. −0.7 mg/L). Improvement in insulin resistance.	Significant histological improvement in the NASH activity index and fibrosis scores.
Bakhshimoghaddam (2018) [39]/Iran	Patients with grade 1–3 NAFLD and aged > 18 years.	History of alcohol abuse, chronic viral hepatitis, autoimmune hepatitis, primary biliary cirrhosis, Wilson disease, history of diabetes, α1-antitrypsin deficiency, hemochromatosis, uncontrolled thyroid conditions, psychiatric disorders, impaired renal function, use of drugs potentially affecting glucose and lipid metabolism, medications that increase the risk of NAFLD, inability to give informed consent, pregnancy, and lactation.	P: 30 (47.1) C: 28 (50) I: 32 (50)/ P: 39.9 ± 10.8 C: 41.1 ± 8.5 I: 38.8 ± 9.0	RCT/Open-label/Placebo/three arm study	Conventional Yogurt	300 g/day of synbiotic yogurt containing *Bifidobacterium animalis subsp. lactis* (*BB-12*) and 1.5 g inulin/24 weeks	Synbiotic yogurt consumption significantly reduced liver steatosis grade and serum levels of ALT, AST, ALP, and GGT compared to conventional yogurt and the control group.	Improvements observed in total cholesterol, TG, LDL, cholesterol, FBS, systolic blood pressure (SBP), liver span, HOMA, QUICKI, total antioxidant capacity (TAC), total oxidant status (TOS), C1q/TNF-related protein 5 (CTRP-5), and glucagon-like peptide 2 (GLP-2) in the synbiotic group compared to other groups.
Sayari (2018) [44]/Iran	With NAFLD, ages 18–60, BMI 25–29.9 kg/m^2^, impaired fasting glucose or impaired glucose tolerance, elevated ALT levels 1.5–3 times above normal.	Alcohol use >10 g/day (women) or >20 g/day (men), other liver diseases (hepatitis B/C), kidney disease, thyroid disorders, immunodeficiency, cholesterol-lowering medication, pregnancy, or breastfeeding.	P: 68 (55.9) I: 70 (64.3)/ P: 43.42 ± 11.65 I: 42.48 ± 11.41	RCT/double-blind/placebo	Maltodextrin capsule as a placebo.	Sitagliptin 50 mg with synbiotic (Familakt containing seven probiotic strains and prebiotic)	Significant improvements in FBS, AST, cholesterol, and LDL levels. Decrease in BMI and weight.	N/A
Mohamad Nor (2021) [50]/Malaysia	Patients aged 18 years and above with an ultrasound diagnosis of fatty liver, a baseline-controlled attenuation parameter (CAP) score measured by FibroScan of >263 dB/m, and a baseline ALT of more than 35 IU/L for males and 25 IU/L for females.	Evidence of other chronic liver diseases (such as hepatitis B or C), autoimmune hepatitis, alcoholic liver disease, acute disorders affecting the liver, drug-induced liver injury, hepatocellular carcinoma, biliary diseases, liver cirrhosis, pregnancy, and recent use of nutritional supplements or lipid-lowering drugs.	P: 22 (77.2) I:17 (64.7)/ P: 52.47 ± 16.73 I: 54.70 ± 10.19	RCT/double-blind/placebo	The placebo contained the same excipients as probiotics but without live bacteria.	Probiotic sachets (MCP^®^ BCMC^®^ strains) containing six different *Lactobacillus* and *Bifidobacterium* species at a concentration of 30 billion CFU.	No changes in hepatic steatosis (as measured by CAP) and fibrosis levels (as measured by FibroScan) between the probiotics and placebo groups. Similarly, no significant changes were observed in liver enzymes, cholesterol, triglycerides, and fasting glucose levels.	Immunohistochemistry analysis showed no significant expression changes in CD4+ T lymphocytes for both groups. However, there was a significant reduction in the expression of CD8+ T lymphocytes and ZO-1 in the placebo group but not in the probiotics group, suggesting that probiotics might stabilize mucosal immune function and protect against increased intestinal permeability.
Barcelos (2023) [12]/Brazil	Adults over 18 years of age with biopsy-proven NASH who had not taken antibiotics or probiotics in the three months prior to enrollment.	Patients with cirrhosis, significant alcohol intake (>15 g ethanol/day), or infections such as HIV, hepatitis B or C. Also excluded were pregnant women, transplant recipients, and patients using immunosuppressants, corticosteroids, valproic acid, tetracycline, amiodarone, or those with other chronic inflammatory diseases or a history of diarrhea.	P: 23 (47.8) I: 23 (34.8)/ P: 51.7 ± 11.9 I: 51.7 ± 11.4	RCT/Triple-blind/Placebo	consisted of a 1 g sachet containing polydextrose/maltodextrin with an identical appearance to the probiotic sachet.	Probiotic supplementation with a mix of *Lactobacillus acidophilus NCFM*, *Lactobacillus rhamnosus HN001, Lactobacillus paracasei LPC-37*, *and Bifidobacterium lactis HN019* at 1 × 10 ^ ^9^ CFU per strain, delivered in 1 g sachets.	No reduce cardiovascular risk markers. Both groups experienced reductions in these markers, but the changes were not significantly different between the probiotic and placebo groups.	No changes in BMI, lipid profiles, or glucose levels in either group. No differences in cardiovascular risk scores.
Escouto (2023) [13]/Brazil	Adult patients with histology-proven non-alcoholic steatohepatitis (NASH) confirmed by liver biopsy within six months prior to inclusion.	Hepatitis B, C, or HIV infection, significant alcohol consumption, decompensated liver disease, hepatocellular carcinoma, use of medications like steroids or vitamin E that could cause steatosis, previous surgeries affecting digestion, total parenteral nutrition, pregnancy or breastfeeding, hypothyroidism, Cushing syndrome, type 1 diabetes, and secondary steatosis causes.	P: 25 (28) I: 23 (13)/ P: 57 I: 58	RCT/Triple-blind/Placebo	Maltodextrin.	Probiotics (*Lactobacillus acidophilus* and *Bifidobacterium lactis*, 1 billion CFUs each).	Improvement in fibrosis markers, though no significant changes were observed in liver enzyme levels, inflammatory markers, or gut microbiota composition.	No changes in metabolic markers (BMI, cholesterol levels, glucose, insulin, HOMA) between groups.
Ayob (2023) [48]/Malaysia	Aged over 18 yeard, diagnosed with NAFLD via ultrasound, CAP score >263, ALT >35 IU/L for males and >25 IU/L for females.	Presence of chronic liver diseases (hepatitis B/C, autoimmune hepatitis, alcoholic liver disease), drug-induced liver injury, liver cancer, biliary disease, cirrhosis, and recent use of nutritional supplements or lipid-lowering drugs.	P: 22 (77.3) I: 18 (66.7)/ P: 49.95 ± 14.05 I: 55.00 ± 11.07	RCT/double-blind/placebo	Identical sachet without probiotic strains.	Multi-strain probiotics (*HEXBIO^®^ MCP^®^ BCMC^®^ strains*, *6 Lactobacillus* and *Bifidobacterium* species).	Reduction in inflammatory cytokines IFN-γ and TNF-α in the probiotic group; no significant biochemical changes in liver enzymes.	No improvements in intestinal permeability markers ZO-1 and zonulin in probiotics versus placebo groups.
28 weeks								
Eslamparast (2014) [32]/Iran	1. Adult patients aged 18 years or older. 2. Diagnosed with non-alcoholic fatty liver disease (NAFLD) based on ultrasound and elevated ALT levels (>60 U/L) for at least 6 months.	1. Viral hepatitis, alcohol use, other chronic liver diseases. 2. Diabetes mellitus, untreated hypothyroidism, systemic diseases, psychiatric disorders. 3. Pregnancy, lactation, or ineffective birth control in women of childbearing age.	P: 26 (42.3) I: 26 (53.8)/ P: 45.69 ± 9.5 I: 46.35 ± 8.8	RCT/double-blind/placebo	Maltodextrin capsules	Synbiotic capsules: *Lactobacillus casei*, L. *rhamnosus*, *Streptococcus thermophilus*, *Bifidobacterium breve*, L. *acidophilus*, *B. longum*, L. *bulgaricus* and fructooligosacch-aride (prebiotic).	Fibrosis scores measured by transient elastography improved significantly in the synbiotic group. No differences in BMI and waist-to-hip ratio between the groups.	Reduction in inflammatory markers (TNF-α, high-sensitivity C-reactive protein, and NF-kB activity). Reductions in liver enzymes (ALT, AST, GGT). Improvement in insulin resistance (HOMA-IR, fasting glucose, and insulin levels).
Mofidi (2017) [42]/Iran	1. Lean patients (BMI < 25) aged 18 or older. 2. Diagnosed with NAFLD using FibroScan (CAP score > 263). 3. Elevated ALT (>60 U/L) for at least 6 months.	1. History of alcohol consumption. 2. Other acute or chronic liver diseases (e.g., hepatitis B or C, biliary diseases) 3. Autoimmune diseases or cancer. 4. Recent use of antibiotics, probiotic supplements, or hepatotoxic medications within 6 months. 5. Pregnant. 6. Patients with more than 10% weight loss during the study.	P: 21 (57.1) I: 21 (52.4)/ P: 44.61 ± 10.12 I: 40.09 ± 11.44	RCT/double-blind/placebo	Maltodextrin capsules	Synbiotic supplementation containing (200 million bacteria): *Lactobacillus casei*, L. *rhamnosus*, *Streptococcus thermophilus, Bifidobacterium breve*, L. *acidophilus*, *B. longum*, L. *bulgaricus*, and fructo-oligosaccharide.	Reductions in hepatic steatosis (FibroScan CAP score) in the synbiotic group compared to the placebo group (*p* < 0.001).	Reductions in FBS and TG (*p* < 0.05). Improvement in ALT and AST. Rreductions in inflammatory markers (hs-CRP and NF-κB) (*p* < 0.05). Improvements in lipid profiles (total cholesterol and LDL cholesterol) were observed.
> 50 weeks								
Duseja (2019) [45]/India	With liver biopsy-proven NAFLD, raised liver enzymes (ALT, AST ≥1.5 times normal) for more than three months, BMI ≥ 25 kg/m^2^, negative for viral and autoimmune liver markers.	Pregnant/lactating women, diabetes mellitus, cirrhosis, recent history of alcohol use above the specified threshold, and use of drugs likely to cause NAFLD (corticosteroids, methotrexate).	P: 20 (75) I: 19 (68)/ P: 33 I: 38	RCT/double-blind/placebo	Identical in appearance and color to probiotic capsules (contained microcrystalline cellulose)	High-potency multistrain probiotic (675 billion bacteria/day, containing eight strains)	Improvements in liver histology, with reductions in hepatocyte ballooning, lobular inflammation, reductions in inflammatory markers and cytokines (TNF-α, IL-1β, IL-6), and NAFLD activity score (NAS).	No weight changes or impact on metabolic syndrome components in either group.
Scorletti (2020) [52]/UK	Adults aged >18 years with proven NAFLD, characterized by liver fat content > 5% as measured by MRS.	History of alcohol consumption exceeding 14 units/week for women and 21 units/week for men, use of antibiotics or probiotic supplements within the last 3 months, history of bariatric surgery, active malignancy, liver disease of other etiologies, or conditions affecting the gut microbiome.	P: 49 (61) I: 55 (69)/ P: 51.6 ± 13.1 I: 50.2 ± 12.4	RCT/double-blind/placebo	Consisted of 4 g/twice a day of maltodextrin (1 capsule a day plus two sachets a day to stir into a cold drink).	Synbiotic treatment consisting of fructo-oligosaccharide (4 g/twice a day) plus *Bifidobacterium animalis* subsp. *lactis BB-12* (10 billion CFU/day, 1 capsule a day).	Significant decrease in ALT, leptin, TNF-α, and endotoxins in the probiotic group compared to placebo.	Weight loss, rather than the synbiotic treatment, was associated with significant reductions in liver fat content and improvements in fibrosis scores. The synbiotic treatment led to changes in specific microbial species but did not influence overall liver health parameters.

ALP: alkaline phosphatase; ALT: alanine aminotransferase; AST: aspartate aminotransferase; BMI: body mass index; C: control; FOS: fructo-oligosaccharides; GGT: γ-glutamyltransferase; HDL: high-density lipoprotein cholesterol; hs-CRP: high-sensitivity C-reactive protein; FBS: fasting blood sugar; FLI: Fatty Liver Index; IBD: inflammatory bowel disease; LDL: low-density lipoprotein cholesterol; LS: liver stiffness; MASLD: metabolic dysfunction-associated liver disease; MRS: magnetic resonance spectroscopy; NAFLD: non-alcoholic fatty liver disease; NASH: non-alcoholic steatohepatitis; P: placebo; I: intervention; TC: total cholesterol; TG: triglycerides.

## Data Availability

All data generated or analyzed during this study are included in this published article.
